# Editorial: The application of neuroscience technology in human assist devices

**DOI:** 10.3389/fnins.2025.1589378

**Published:** 2025-03-25

**Authors:** Yining Hou, Yue Ma, Shuang Liang, Yisi Liu, Zirui Lan

**Affiliations:** ^1^Shenzhen Institute of Advanced Technology, Chinese Academy of Sciences (CAS), Shenzhen, China; ^2^Nanjing University of Posts and Telecommunications, Nanjing, Jiangsu, China; ^3^Fraunhofer, Singapore, Singapore

**Keywords:** neuroscience technology, robot, human assist devices, EEG, sEMG, algorithm

Advancements in sensor technology have narrowed the gap between acquiring neural signal data and applying it to control human-assist robots. Walking assistive devices represent a key focus in exoskeleton robotics, potentially aiding patients with mobility challenges and seniors at risk of falls. Zhou et al. conducted a study wherein they collected surface electromyography (sEMG) features related to gait characteristics using a soft robotic exoskeleton. This methodology investigated the prediction of fall risks in patients diagnosed with cerebral small vessel disease (CSVD) via 12 predictive models. The finding emphasizes the critical necessity of considering gait characteristics, electromyographic features, baseline data, and imaging markers in the early identification of fall risk. Another investigation by Chen et al. regarding fall risk established an interpretable multimodal time-series (iP3T) model. This framework is designed to enhance the prediction of gait phases in wearable technology exoskeletons. The pivotal findings from this research correlated the data points with the existing gait phases during interventions, employing both biomechanical and neuromuscular signals. This approach yields more comprehensive and precise predictions of gait phases, thereby facilitating improved risk assessment and intervention strategies. Wang et al. conducted a systematic investigation on surface electromyography (sEMG) signals obtained from specific positions of the lower limb musculature, which aimed at classifying muscle fatigue. The diverse environmental conditions in which wearable robotics are deployed introduce significant complexities in the dynamics of movement. This study proposes a transfer learning approach that excels in its capacity to generalize across varying physical contexts, thereby serving as a valuable instrument for adaptation to novel environmental scenarios.

Accurate data acquisition is crucial for advancing human assistive devices. Virtual reality technology enables the precise tracking of electroencephalogram (EEG) signals by reducing the interference of various external factors, particularly in the area of human-brain interactions. Liu and Zhao introduced the EEGMind-Transformer, a deep learning architecture that addresses the challenge of the intricate spatiotemporal relationship in EEG data more effectively than conventional approaches. Their results yielded an accuracy of 92.5%, an F1-score of 90.8, and an AUC of 94.2% across various datasets. A key goal of this article is to understand the brain functions associated with mental disorders, working toward mental health monitoring. The EEGMind-Transformer model possesses the potential for deployment in wearable electroencephalogram (EEG) devices, facilitating the monitoring of stress and the early detection and tracking of mental health trends. This advancement may yield critical insights into biomarkers derived from EEG-based assessments. The model exacts temporal dependency, models the brain's hierarchical structure, and captures the interactions among brain regions; it also synthesizes spatial and temporal elements to generate the representations. The principal limitation resides in the dependency on high-quality, artifact-free electroencephalography (EEG) data. The process of eliminating noise in real-world clinical environments presents considerable challenges. In this context, Lakshminarayanan et al. conducted an investigation into the synergistic effects of virtual reality (VR)-based action observation and kinesthetic motor imagery (KMI). Their objective was to enhance motor imagery capabilities through the integration of VR and EEG methodologies. The study hypothesized that the combination of VR-based action observation and KMI significantly enhances patterns of brain rhythmic activity and improves task differentiation compared to KMI executed in isolation, devoid of action observation and subjected to high noise levels. Another manuscript by Simar et al. explored the application of virtual reality (VR) technology for hand pose classification. A VR headset equipped with advanced hand-tracking capabilities to facilitate the simultaneous recording of electromyography (EMG) signals and hand pose estimations. By employing a neurophysiological integration method to delineate the phasic and tonic components of the EMG signals, the findings demonstrated potential enhancements in posture recognition. The authors posited and subsequently validated that their machine learning models would enhance the control of myoelectric prostheses. Furthermore, EEG and brain-computer interfaces (BCI) represent promising avenues for facilitating communication in patients with communication impairments. LaRocco et al. designed their experiment utilizing a lexicon comprised of English words, integrating 44 distinct phonemes, each corresponding to a specific EEG pattern. The model attained a mean accuracy of 97 ± 0.001%, with a mean F1 score of 0.55 ± 0.01 and a mean area under the receiver operating characteristic curve (AUC-ROC) of 0.68 ± 0.002. This advancement in phoneme-based EEG BCI can manifest as either hardware or software solutions, illustrating the potential for this technology to be implemented in real-world applications. The biggest limitation of this work lies in the lack of real-time feedback, as the trial length of 1 second exceeds human awareness and neural processes for lexical selection. This indicates some future directions and obstacles that could arise overcome.

In conclusion, the use of electroencephalogram (EEG) and surface electromyography (sEMG) for acquiring neural activity and peripheral nerve activity has become popular due to their non-invasive manner, relatively low cost, portability, and easy implementation.

